# Establishing nurse-led active surveillance for men with localised prostate cancer: development and formative evaluation of a model of care in the ProtecT trial

**DOI:** 10.1136/bmjopen-2015-008953

**Published:** 2015-09-16

**Authors:** Julia Wade, Peter N Holding, Susan Bonnington, Leila Rooshenas, J Athene Lane, C Elizabeth Salter, Kate Tilling, Mark J Speakman, Simon F Brewster, Simon Evans, David E Neal, Freddie C Hamdy, Jenny L Donovan

**Affiliations:** 1 School of Social and Community Medicine, University of Bristol, UK; 2 Nuffield Department of Surgical Sciences, University of Oxford, Churchill Hospital, Oxford, UK; 3 Department of Urology, Musgrove Park Hospital, Taunton and Somerset NHS Foundation Trust, Taunton, UK; 4 Department of Urology, Churchill Hospital, Oxford University Hospitals NHS Trust, Oxford, UK; 5 Department of Urology, Royal United Hospitals Bath NHS Foundation Trust, Bath, UK; 6 University Department of Oncology, Addenbrooke's Hospital, University of Cambridge, UK; 7 NIHR Collaboration for Leadership in Applied Health Research and Care West at University Hospitals Bristol NHS Trust, Bristol, UK

**Keywords:** UROLOGY

## Abstract

**Objectives:**

To develop a nurse-led, urologist-supported model of care for men managed by active surveillance or active monitoring (AS/AM) for localised prostate cancer and provide a formative evaluation of its acceptability to patients, clinicians and nurses. Nurse-led care, comprising an explicit nurse-led protocol with support from urologists, was developed as part of the AM arm of the Prostate testing for cancer and Treatment (ProtecT) trial.

**Design:**

Interviews and questionnaire surveys of clinicians, nurses and patients assessed acceptability.

**Setting:**

Nurse-led clinics were established in 9 centres in the ProtecT trial and compared with 3 non-ProtecT urology centres elsewhere in UK.

**Participants:**

Within ProtecT, 22 men receiving AM nurse-led care were interviewed about experiences of care; 11 urologists and 23 research nurses delivering ProtecT trial care completed a questionnaire about its acceptability; 20 men managed in urology clinics elsewhere in the UK were interviewed about models of AS/AM care; 12 urologists and three specialist nurses working in these clinics were also interviewed about management of AS/AM.

**Results:**

Nurse-led care was commended by ProtecT trial participants, who valued the flexibility, accessibility and continuity of the service and felt confident about the quality of care. ProtecT consultant urologists and nurses also rated it highly, identifying continuity of care and resource savings as key attributes. Clinicians and patients outside the ProtecT trial believed that nurse-led care could relieve pressure on urology clinics without compromising patient care.

**Conclusions:**

The ProtecT AM nurse-led model of care was acceptable to men with localised prostate cancer and clinical specialists in urology. The protocol is available for implementation; we aim to evaluate its impact on routine clinical practice.

**Trial registration numbers:**

NCT02044172; ISRCTN20141297.

**Strengths and limitations of this study**

- The strengths of this study include that it was embedded in the Prostate testing for cancer and Treatment (ProtecT) randomised controlled trial of treatments for localised prostate cancer, providing a framework for the development of a model of nurse-led active monitoring/active surveillance care and enabling the training of nurses alongside clinicians.
- It was supplemented by data from another large nationally funded study that enabled views on nurse-led active monitoring/active surveillance care for men with localised prostate cancer from outside the ProtecT trial to be accessed.
- The model of care has been clearly outlined and is ready to be evaluated.
- The limitations of the study are that the model has been presented and evaluated descriptively; it has not yet been fully evaluated for its cost-effectiveness or impact on routine practice.

## Introduction

Prostate cancer (PCa) is the most common cancer in men in the UK, making up 26% of male cancer diagnoses in England and Wales in 2010.^1^ The 2010 UK PCa prevalence was estimated at over 250 000 with the possibility of a threefold increase or more by 2040.^2^ Given the indolent nature of many of these cancers and the risks of side effects associated with current radical treatments,^3^ various non-radical approaches have been developed for men diagnosed with localised disease.^4^ ^5^

Active surveillance (AS) and active monitoring (AM) programmes aim to offer men the option of avoiding immediate surgery or radiotherapy (RT; and their adverse events) with disease monitoring, so that those whose disease remains stable can avoid intervention and those whose disease progresses can have curative treatment. Programmes have developed with variable eligibility criteria, management protocols and triggers for clinical review and change of management.^6^ AS was developed originally in North America^7^ and is now recommended in European^8^ and US^9^ guidelines. UK National Institute for Health and Care Excellence (NICE) guidelines recommend that AS be offered to men suitable for radical treatments and with low-risk localised PCa (defined as prostate-specific antigen (PSA) ≤10 ng/mL and Gleason ≤6 and stage T1-T2a disease); and considered for men with intermediate-risk localised PCa (defined as low risk but with one or more of: PSA=10–20 ng/mL or Gleason 7 or T2b disease).^1^ Monitoring in AS programmes tends to include scheduled repeat prostate biopsies as well as regular PSA testing and digital rectal examination (DRE).^1^ The AM protocol for the Prostate testing for cancer and Treatment (ProtecT) trial included men with low-risk and intermediate-risk PCa and employed a particular form of regular PSA monitoring (see below). It differed from AS programmes primarily as prostate biopsies were not part of regular scheduled monitoring but could be used as part of the clinical assessment of disease progression.^10^

Once on AS/AM, men need regular monitoring. Although UK NICE guidelines allow for PSA testing to take place in primary care and for DRE to be carried out by a suitably qualified health professional,^1^ monitoring is usually led by urologists, which results in increased demands on urology services, filling clinics with routine management and infrequent interventions rather than expert care.^11^ Alternatives to standard consultant-led outpatient clinics include clinics led by specialist nurses. Features that distinguish nurse-led clinics include the nurse having their own patient caseload, an increase in autonomy of the nursing role, and the ability to admit and discharge patients from the clinic or to refer to other clinical colleagues.^12^ Nurse-led management is well established in cancer care,^13^ including post-treatment follow-up in urological oncology,^14–17^ and shared care, integrated services between primary and secondary care.^18^ However, nurse-led clinics for routine monitoring postdiagnosis are not yet common.

### Description of nurse-led ProtecT AM

The aim of nurse-led ProtecT AM was to ensure that consistent delivery of routine activity was achieved by nurses, with rapid provision of consultant urologist advice and support when required. The protocol for nurse-led ProtecT AM therefore included guidelines for nurses and established clear lines of communication between the nurses and principal study urologist.^10^ There were four core components: (1) the treatment pathway that defined the type and timing of tests, significance of test results and actions required; (2) quality assurance processes, including standard operating procedures (SOPs), training, meetings and site monitoring;^19^ (3) development of integrated working with primary care for PSA testing and (4) feedback from patients regarding timing, frequency and location of clinics. All four core components were incorporated in nurse-led clinics.

The ProtecT AM protocol involved regular PSA tests with referral to the urologist for additional tests (eg, prostate biopsy, bone/MRI) if there were indications of progression or other clinical concerns. At the time of each PSA test, the most recent PSA result was compared with previous results and in particular results taken 12 months previously. Patient-reported physical problems, in particular lower urinary tract symptoms and psychological concerns, were also considered. Whenever possible, PSA testing was performed in primary care, 2–3 weeks prior to the clinic appointment date, allowing time for a nurse-led preclinic review of patient history and current status and additional tests as necessary. Careful tracking and coordination of appointments, using a database with built-in alerts, helped ensure optimum efficiency, particularly with regard to face-to-face appointments.

AM appointments were conducted by research nurses all of whom underwent initial and ongoing local and national training, including one-to-one assessment of practice within the trial. Communication within the nursing team was shared, so that each nurse was able to provide planned and short-notice cover. All nurses had direct access to the principal study urologist and other designated urologists and oncologists to ensure timely, expert advice. Documented permission for nurses to deliver treatment was officially delegated by the principal urologist following review and approval of the RCT by the host National Health Service (NHS) trust. Patients had direct access to the ProtecT trial AM team by telephone (24/7 answerphone available) or by email. The location of face-to-face appointments in either primary or secondary care took account of patients’ wishes, availability of staff and the level of expertise required for any given appointment. Telephone appointments were conducted when a face-to-face appointment was not required, for example, interim PSA test results.

This paper presents the model of nurse-led AM that was developed within the ProtecT randomised controlled trial (RCT) of treatments for men with clinically localised PCa. A formative evaluation of its acceptability to patients, clinicians and nurses within and outside the trial suggests that it could be implemented more widely to reduce pressure on increasingly overstretched urological services.

## Methods

### The ProtecT study

The ProtecT RCT involved a programme of PSA testing among men in the community aged 50–69 years in nine trial centres.^10^ Participants diagnosed with clinically localised PCa (T1-T2, PSA 3.0–19.99 ng/mL, Gleason 6 or 7) were offered randomisation to AM, three-dimensional conformal external beam radical RT or radical prostatectomy. ProtecT trial centres were led by a consultant urologist and a senior research nurse. In total, 545 men were randomised to AM; in addition 529 refused randomisation and chose AM.^10^ All patients were followed up with the same protocol.

A nested trial during the feasibility phase of the trial showed that nurses were as effective, and more cost-effective than doctors for recruitment.^20^ Nurse-led clinics and follow-up procedures were developed, likewise led by trained research nurses, with the aim of ensuring high levels of protocol adherence, data quality and minimising loss to follow-up.^10^

The protocol for the AM pathway was developed for the trial based on available evidence.^21^ Scheduled rebiopsy was not included, and disease review was based on a confirmed 50% rise in PSA level over the previous 12-month period.^10^ As the trial progressed, the protocol was reviewed by the international Trial Steering Committee (TSC) to ensure continued relevance, and SOPs were developed and discussed regularly at meetings and training events. All amendments to the protocol received ethics committee approval.

Over 1000 men in nine clinical centres across the UK received ProtecT nurse-led AM, including those randomly allocated to AM and those who chose AM having declined randomisation. Recruitment to the ProtecT main trial started in 2001 and ended in 2009.^10^

### Evaluating the acceptability of nurse-led ProtecT AM

#### Participants and methods

The aim of this study was to assess the acceptability of ProtecT nurse-led AM to men, urologists and research nurses within the ProtecT trial, and to compare these with experiences of standard urologist-led AS care in urology centres outside the ProtecT trial. The study combined in-depth interviews and questionnaire surveys.

*Patients.* As part of a larger ProtecT qualitative study, 24 men randomised to or choosing ProtecT AM in four clinical centres were invited for interview. Purposive sampling was used to include men across the age and socioeconomic range with low-risk and intermediate-risk localised PCa at diagnosis (Gleason score 6 or 7 and PSA ≤19.99 ng/mL) who had undergone AM for at least 6 months. These men were informed of the interview study by letter and patient information leaflet, followed by a telephone call by JW 2 weeks later, unless men had indicated refusal using a written reply. Interviews were conducted by JW or CES (credentials in online supplementary appendix C), during which views on nurse-led AM were explored. In a related study exploring urologist-led AS practice in three centres not involved in the ProtecT trial,^22^ purposive sampling was used to identify men across the age and socioeconomic range with low-risk localised PCa at diagnosis (Gleason score 6 and PSA ≤10 ng/mL) who had undergone AS for at least 12 months and were judged by their clinician to show no evidence of disease progression. Twenty men were given or sent a recruitment information pack which asked them to contact LR if they wished to participate in an interview with LR or JW about their views of AS.

*Health professionals*: All 12 urologists and 3 clinical nurse specialists (CNSs) delivering consultant-led AS in three clinics not involved in the ProtecT trial were invited for interview with LR or JW to discuss their views about AS.^22^ All 11 urologists and 23 research nurses currently working in the ProtecT trial were invited to complete questionnaires about their experiences of delivering nurse-led AM.

Twenty-two of the 24 invited men took part in interviews investigating experiences of nurse-led AM within the ProtecT trial (AM1–AM22, table 1, two declined participation without stating the reason). All 20 invited men receiving AS outside the ProtecT trial consented to participate in single interviews of their experiences (AS1–AS20, table 1). Some men chose to be interviewed with their wife or partner present, in which case these were subject to the same consent procedures. All 12 urologists (U1–U12) and all 3 CNSs (CNS1–CNS3) invited to single interviews of their experience of urologist-led AS outside the ProtecT trial agreed to participate. No interviewees were previously known to their interviewer. All participants were informed of their interviewer's occupation, employer and the purpose of the interview.

**Table 1:** Characteristics of men participating in the in-depth interview study

	Men receiving AM within ProtecT (N=22)	Men receiving AS (N=20)
Age at time of first interview: mean (range)	64;7 (53;11–70;10)	65 (55–70)
Ethnicity, N (%)		
White	21	20 (100)
Other	1	0 (0)
Treatment decision-making		
* *Accepted randomly allocated treatment	12	NA
* *Chose treatment	10	20
Interview type*		
Telephone	33	0
Face-to-face	40	20

*Some men in ProtecT gave several interviews.

AM, active monitoring; AS, active surveillance; NA, not available; ProtecT, Prostate testing for cancer and Treatment.

#### Data collection

**Figure 1:**
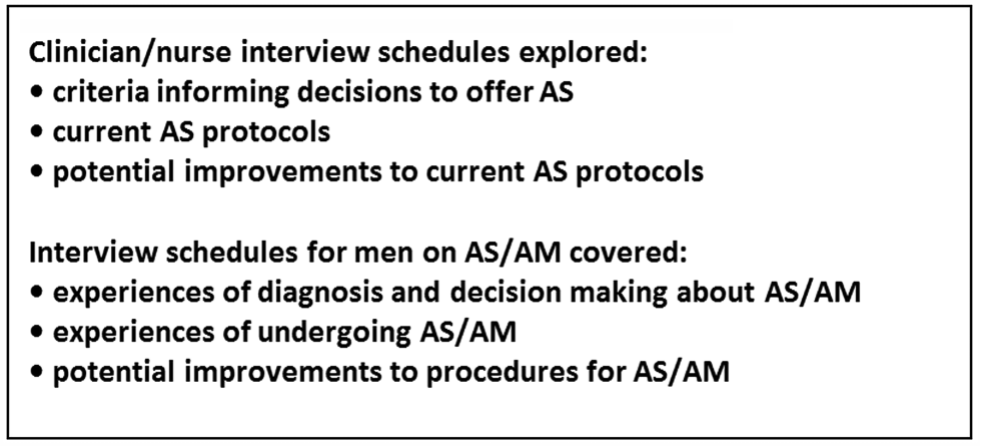
Topics covered in in-depth interviews (AM, active monitoring; AS, active surveillance).

Interviews were semistructured using a topic guide (figure 1) to elicit interviewees’ experiences of AS/AM and reflections on how procedures could be improved, while allowing interviewees to raise issues of importance to them. Interviews were face-to-face or by telephone in each interviewee's preferred location (eg, home or clinic room in urology centres) and ranged from 8 min (ProtecT patient, third interview) to 1 h and 36 min (patients), with most lasting between 30 and 60 min; and from 20 min to 1 hs and 5 min (health professionals). Findings from patient and health professional interviews were used to develop urologist (see online supplementary appendix A) and nurse (see online supplementary appendix B) questionnaires. These were completed anonymously and returned via a clerical officer to maintain confidentiality. A reminder was sent after 4 weeks to request completion and return of the questionnaire.

#### Qualitative data analysis

Interviews were audio-recorded, transcribed verbatim and transcripts supplemented by accompanying field notes. Transcripts were scrutinised to identify recurrent themes emerging from the data. Transcripts were analysed in groups (eg, men receiving nurse-led vs urologist-led care, urologists vs specialist nurses, by study centre) to identify commonalities and contrasts between groups or individuals. The interview topic guides for each qualitative study were refined during the interview process as themes emerged, in accordance with principles of constant comparison methods derived from grounded theory.^23^ ^24^ Data collection and analysis proceeded in tandem, with sampling continuing until the point of data saturation. Analysis was facilitated by use of qualitative computer software NVivo,^25^ and led by JW, with a subset of 32 interviews also analysed by LR. Findings were discussed and synthesised to maximise reliability of coding and data interpretation.^23^

#### Questionnaire data analysis

Questions were presented using a mixture of dichotomous ‘yes’ or ‘no’ responses, a five-point Likert scale (ranging from ‘strongly agree’ to ‘strongly disagree’), tick boxes for named activity and free text boxes. Descriptive analyses were undertaken because of the small number of participants in each survey. Free text comments were analysed using thematic analysis.^23^

## Results

### Interview study

The majority of participants in the ProtecT trial reported satisfaction with nurse-led AM. Three key themes emerged in support of nurse-led care: (1) efficient use of resources, (2) quality of care and (3) convenience of care. A majority of men receiving consultant-led AS (N=12) and a majority of clinicians or nurses involved in delivering AS outside the ProtecT trial (N=9) supported alternatives to the consultant-led model, citing the reasons above and also suggesting that (4) nurse-led care was a natural extension of existing nurse practice. A small number of concerns were raised about nurse-led care (5), but suggestions were made for key components of care (6) that would minimise risks when moving to a nurse-led model.

Several ProtecT trial participants were interviewed more than once therefore interview number and timing postdiagnosis is given following each quote, for example, AM1/2, 2 years 3 months denotes second interview with participant AM1, 2 years, 3 months postdiagnosis.

#### Efficient use of resources

Men receiving both nurse-led and consultant-led care argued that nurses could provide more cost-effective care than consultants:

Why bring him [urologist] in at enormous expense when he's far better cutting people up and sorting them out? (AM6/2, 2 years 1 month)

I'm very concerned about the cost to the NHS. Anything that reduces the cost of the NHS I'd be happy about. And if it frees up a consultant, or whatever, to do something else. (AS12)

Urologists and CNSs delivering consultant-led AS agreed that most appointments were routine and could be safely delegated to CNSs to maximise efficiency in the face of growing numbers of men on AS/AM:

We're just getting inundated by people, and there's a limit to the number of consultants that the hospital and the country can afford. (U9)

It's something that can be quite clearly protocolised and be run safely by a clinical nurse specialist. (CNS1)

#### Quality of care

Men receiving nurse-led AM reported high levels of confidence in the quality of care delivered by nurses. This confidence developed over time and was attributed to how nurses responded to concerns or queries:

Initially I was a bit concerned about it to be honest with you. As I say, initially—we're talking 2008 now—I was seeing a nurse rather than a urologist. But over the time and especially with [NURSE NAME] I began to believe that they have such a base of knowledge that I have confidence in what they are saying. (AM3/3 5 years 11 months)

These men reported that nurses (1) gave men enough time to talk through concerns, (2) were easy to contact, (3) dealt promptly and reassuringly with concerns, (4) were knowledgeable and experienced and (5) worked effectively as a team with other team members, in particular arranging further investigations or consultations with urologists as required:

I think it's just very caring, understanding, knowledgeable, you know, half an hour each time I go. (AM8/2, 2 years 1 month)

They do just ask how do you feel and whether it's giving you any trouble. When I do go over to [HOSPITAL NAME] if I am a little worried and I do talk to them, they put me at my ease. (AM12/2, 2 years 2 months)

I find the whole thing so far very helpful and very reassuring. I can't highlight anything where they have been unhelpful, I have never left thinking ‘I wish I had known more a bit more about this or that…I had a full body scan and thank goodness that proved to be no problem and no cause for concern. So again, I have got the confidence that if I can explain that I am having a problem in one respect or another, she [RESEARCH NURSE] will do her best to get it sorted. (AM3/3, 6 years 0 months)

Men needing to see a consultant for review explained further benefits:

It's nice to have the nurse there [during consultation with urologist] perhaps so you can defer the decision and have a private conversation with the nurse as it were afterwards…perhaps you don't think of the questions while you're in and the questions form in the back of your mind which you wished you had asked. (AM9/2, 2 years 2 months)

It's this lack of consistency [under consultant-led care]; you don't see the same person. (AS6)

I think if you're a patient yourself you would always want to see the same person, because you feel that you build up a rapport with them. (CNS1)

Nurse-led care was perceived as potentially more holistic:

I think it was interesting that it [ProtecT] was a nurse-led study…consultants can be very focused on your specific condition and what they can do for that and I think that a nurse can be much more holistic. (AM18/1 6 years 7 months)

I think also we're very good, as CNSs, at giving out the information of living with the disease, eating well, keeping fit, keeping active and all—I think they'd get a more holistic service if it was done by the CNSs. (CNS1)

#### Convenience of care

Men receiving ProtecT AM valued the convenience of deciding where their PSA blood test was taken, how they received their result and how frequently PSA tests were conducted. They had PSA tests at their general practitioner (GP) practice (N=15), urology centre (N=6) or during home visits by a community matron caring for a partner (N=1). Some received results from ProtecT nurses during face-to-face appointments in urology centres or by telephone; others attended their GP practice and contacted practice staff for results. Men chose the option to suit them best:

As long as I'm happy to do the phone thing then that's fine, it suits me. Its 20 miles isn't it, it's not an easy place to get into town in the morning, for 9 o'clock or half past 9. It's better now because the car parking is a bit better than it was but initially it was a bit of a pain because I needed somewhere to park up. Yeah, so it's once a year going down and that's fine. (AM7/3, 4 years 9 months)

Men receiving urologist-led AS (12/20) suggested that alternatives to regular urologist-led review (such as by telephone or Skype) would offer more convenient care. Men receiving both models of care reported reluctance to travel to urology centres because of lost time, travel and parking costs. Such costs were difficult to justify if an appointment was brief, routine and involved no physical examination:

It cost me nearly four quid for a five minute appointment when I actually got in there. (AS2)

Partner There were one or two appointments that we came to where we thought, ‘Well’ ‘Could have done that over the phone’. (AS11)

Even those who explicitly valued urologist-led care felt face-to-face contact could be reduced for mutual benefit:

I think it's quite good to see the people who may ultimately operate on you or whatever happens to be the case, and have that dialogue with them[…] and they [men] would only come in if they [urologists] were concerned about the velocity or whatever happened to be the case. (AS9)

#### Extension of current practice

Urologists outside the ProtecT trial reported a desire to set up nurse-led clinics, and some already had specialist nurses who could take on such a role:

One of the things that we are looking at doing, as our CNS numbers have now improved, is to set up a nurse-led clinic for men on AS…All the patients that have radical prostatectomies or brachytherapy, they're seen in the nurse-led clinic, and they don't see the consultants, and they report very high levels of satisfaction. (CNS1)

I think there is a bit of sharing. I've trained a specialist nurse to see these patients and she runs with that guideline that we've mentioned. I trained her to do rectal examinations and to use an online calculator for doubling time. And she tends to do the clinics when I'm doing other clinics so she can pop in and ask advice if necessary. (U11).

#### Concerns about nurse-led care

Some concerns were voiced about nurse-led care. All men perceived urologists to be the overall experts and believed key decisions should be made in consultation with them:

I would probably have been more reassured if (CONSULTANT NAME) could have seen me…I mean if he had my case notes in front of him and he said ‘Look I don't think it is opportune at the moment to do any operations’ I would feel more reassured…yeah that would be more helpful to me…I think at some stage I would probably like to say, ‘Let me have a word with (CONSULTANT NAME)’ you know? ‘Let me see the top honcho here and see what he says about it all’. (AM6/2 5 years 0 months)

Some men receiving urologist-led care (N=3/20) argued that, for this reason, care exclusively by the urologist was preferable:

You want to be in the business of talking to the people who'd be doing the business [surgery] on you if it comes to the crunch. (AS9)

In contrast, the two men receiving nurse-led care (AM3 and AM6) who expressed similar initial concerns about receiving nurse-led care as opposed to consultant-led care reported these had dissipated entirely by the time of their third interview (5–6 years postdiagnosis).

Three negative experiences of nurse-led care emerged. Two men receiving nurse-led care reported receiving conflicting advice about the need to switch to radical treatment, and another believed a nurse overreacted to a single rise in PSA levels by suggesting a switch to radical treatment, whereas the urologist subsequently reassured him there was no such need. A specialist nurse likewise suggested nurses might be more cautious:

It was confusing. Nobody was saying ‘yeah’ and well it was half and half like you know and getting mixed vibes. That was the only thing that I found wrong with it all (AM4/3, 5 years 6 months)

So the difference between nurse specialist follow-up and consultant follow-up is that the nurse specialist will probably be more cautious. (CNS2)

Such reactions might be expected when following a protocol and would need to be balanced with good team working.

#### Key components of nurse-led care

Men receiving both models of care and urologists/CNSs identified a number of essential components for successful nurse-led care. All stated that nurse-led AS must be explicitly protocol-driven, with frequency of routine PSA tests, routine DRE or routine rebiopsy clearly defined and clear guidelines in place to determine when men should be referred to urologists for consultation or further investigation:

That's one of the things that we'd have to iron out as a department, is exactly the protocol for repeat digital rectal examinations. (CNS2).

It's good to get things into protocol driven things…So we could trust our nurse specialists to, you know, tell us if they fall outside. (U9)

Effective communication between nurses and urologists was essential and patients believed endorsement of the CNS’ skills by the urologist was also important:

I'm aware that [NURSE NAME] has built a good rapport with the consultants, so therefore we're getting the best people… AM9/3

Men wanted reassurance that delegation of routine PSA blood tests to nurses should be conditional on nurses working under direction of the urologist, with rapid pathways for referral when required:

I still want the idea that the person can refer you…to the consultant, should they feel it necessary. AS12

### Questionnaire survey in the ProtecT trial

All 11 urologists and 23 research nurses surveyed in the ProtecT trial completed and returned the questionnaire (100% response rate). Urologists and nurses all agreed (100% of nurses and 51% of urologists strongly agreed) that the AM clinics provided benefits to patients including psychological support, continuity of care, flexibility of appointments, good quality of care and reduced the burden on the NHS. Almost all urologists (N=9) and nurses (N=19) agreed that AM allowed nurses to refer concerns to relevant clinicians, and most urologists (N=8) and nurses (N=18) believed AM clinics gave nurses the opportunity to initiate appropriate diagnostic tests. All urologists agreed that nurse-led AM clinics provided reliable and timely appointments for the participants, with seven believing they were likely to be cost-effective. Just over half the urologists (N=6) carried out or arranged routine diagnostic tests themselves. All nurses felt they had acquired specialist skills as a result of being involved in AM clinics and reported job satisfaction. The only area where there was a lower level of consensus was in relation to nurses being able to admit patients to hospital: 9 (39%) indicated they could, 4 (17%) said they could not and 10 (43%) were unsure or did not respond.

Free text comments by urologists showed some wished to extend nurse skills to enable them to carry out DRE and TRUS-Bx investigations, but were aware that appropriate training, knowledge and support would be needed. DRE was the most frequently cited skill that nurses wanted to acquire and current inability to do this was cited as a significant gap in current nurse-led AM practice.

Research nurses indicated that nurse-led AM had enabled them to develop greater autonomy, particularly with regard to frequency of PSA testing and how patient appointments were organised. Despite the protocol allowing nurses to initiate testing, five nurses commented that they continued to make decisions about testing in consultation with the urologist; some variation in nurses initiating tests or hospital admission was attributed to local hospital trust policy. Some potential stressors were identified, arising from lack of training or support to carry out extended duties, but these were few in number and could be countered by good support from urologists. Nurses believed that AM was popular with patients who informed them that they valued the continuity of care, accessibility and availability of staff. They considered that the high percentage of men continuing in AM was evidence of this.

## Discussion

### Description of main findings

The ProtecT trial developed a model of nurse-led AM for men with localised PCa that was acceptable to men, urologists and nurses within the trial and of interest outside it. Urologists believed that nurse-led AM had enabled high-quality care to be delivered in a way that simultaneously reduced the burden on urologist clinics and brought patient benefits. Nurses believed nurse-led AM enabled them to increase their autonomy and develop professionally, while providing a high-quality, flexible service to patients. Nurses valued good communication with urologists and used this to support their practice even when they had autonomy to act (eg, in initiating tests). Patients within the ProtecT trial were very positive about nurse-led care because it allowed flexibility about the frequency and location of PSA blood tests and appointments to discuss PSA results, and was perceived to offer care that was more holistic, more accessible and had greater continuity than could be achieved through the urologist-led model. Patients outside the ProtecT trial perceived nurse-led care to be acceptable and desirable given the imperative to use NHS resources cost-effectively, and on condition that there were clear lines of communication between nurses and urologists. Small numbers of men receiving consultant-led care outside ProtecT (3/20) expressed a preference for care from the urologist; a similar number receiving nurse-led care within ProtecT (2/22) expressed similar preferences at the time of first interview (6–12 months postdiagnosis), but these preferences had been replaced with preference for nurse-led care by the time of the third interview (5–6 years postdiagnosis). Urologists and CNSs outside the ProtecT trial advocated a move towards nurse-led AS/AM, believing it offered more efficient use of resources, a means to maintain and improve the quality and consistency of care and was a natural extension of existing nurse-led practice in monitoring men who had received radical treatments. Nurses and urologists highlighted the need for a clear protocol to be in place, for clear lines of communication to exist between nurses and urologists, and for nurses to be given the appropriate skills, knowledge and support to meet the demands of the role. Where tasks such as DRE were not yet undertaken by nurses, urologists and nurses wanted to expand their practice to include DRE as part of routine care, but were aware that training and support was required for them to do so.

### Limitations of study

This research did not attempt to evaluate the clinical effectiveness of the ProtecT nurse-led AM care model; publication of the ProtecT trial findings is due in 2016. The ProtecT AM nurse-led care was developed and evaluated within the ProtecT RCT, with support from National Institute for Health Research (NIHR) for research nurses to undertake follow-up and participate in national meetings, site monitoring and training. There was a stable workload in each trial centre once recruitment had ceased, whereas in routine clinics the number of men will continue to increase, potentially requiring streamlining of the protocol over time.

The ProtecT RCT provided a framework to develop a consistent protocol with application and evaluation in nine UK cities. The protocol is clearly structured but flexible enough to encompass the particular model of AS/AM that urologists wish to follow. There will be continued pressure on urologist-led AS care as numbers of men receiving PSA tests continue to increase following the introduction of the latest NICE guidance.^1^ The ProtecT protocol would enable more men to be monitored by expanding the role of CNSs and with further integration with primary care. Although the ProtecT model of nurse-led AM allowed for PSA testing at GP surgeries, this study did not investigate GPs perceptions of nurse-led care or GPs views on nurse-led shared care delivered via GP surgeries; further evaluation is required to identify implications of greater integration with the primary care setting and the challenges of a shared care protocol.

Although urologists, nurses and patients believed nurse-led clinics were and would be cost-effective, this requires evaluation; nurse-led recruitment was cost-effective.^20^ The ProtecT trial employed an IT system that flagged men and produced reports to guide timing of follow-up; routine clinics would benefit from comparable IT support. There was some variation in clinical practice by centre in terms of what activity the urologist and nurse took responsibility for, hence variation in urologist/nurse responses to questionnaires, particularly in relation to tests initiated out by nurses.

### Relationship of findings to existing literature

Previous research has demonstrated the acceptability to patients of nurse-led care for cancer survivors generally^13^ as well as PCa survivors following radical treatments in particular.^14^ ^16^ Nurse-led care has also been evaluated for PCa diagnosis^26^ ^27^ with positive findings regarding patient satisfaction. This study described the development of a model of nurse-led care for men with low-risk or intermediate-risk PCa and evaluated its acceptability to patients, clinicians and nurses, sampled to include experiences of both urologist-led and nurse-led care. Much previous work evaluating nurse-led care for PCa has focused on follow-up either immediately postdiagnosis^14^ or postradical treatment;^17^ this study included experiences of patients and staff involved in AS/AM for up to 13 years.^10^ Previous research highlighted the need for clearly defined levels of responsibility and triggers for onward referral;^17^ this study confirmed these findings. Previous research specifically evaluating nurse-led care for monitoring men with PCa delivered care exclusively via telephone,^14^ with face-to-face and telephone groups giving comparable ratings for general satisfaction and professional care, and men receiving telephone-led care having a shorter waiting time and lower scores for depth of relationship and perceived time with the nurse.^14^ In the ProtecT trial, there were high levels of satisfaction with nurse-led care which facilitated care tailored to the individual. This tailoring of care may have countered some of the loss of satisfaction associated with exclusively telephone follow-up.

### Implications of study for policymakers, clinicians and nurses, and future research

This study demonstrated that nurse-led care had the approval of patients, urologists and nurses inside and outside the ProtecT trial. Reduction of routine tasks by consultants would suggest likely cost-effectiveness if urologists move to more specialised tasks. Concerns about the psychological implications of AS/AM are sometimes expressed^28^ and although evidence indicates patients undergoing AS/AM report good quality of life, more data are needed on longer follow-up.^29^ Accurate clinical information is important in order to inform the patient about the current status of their PCa, but men in the interview study appreciated the holistic care and interest in their individual needs, circumstances, beliefs and values provided by nurses, backed by specialist urologists. The model of nurse-led care allowed considerable flexibility in tailoring how, where and how often PSA tests were carried out and this flexibility was highly valued by ProtecT trial patients. Further research will be required to establish whether this degree of flexibility is cost-effective.

Nurses will need to be trained to take on the additional responsibility and skills required for nurse-led AS/AM. The opportunity to run nurse-led clinics in parallel with urologists was highly valued by nurses and urologists; further work is needed to establish how best to build these into clinical practice and whether such parallel clinics are required during nurse skills acquisition or are of value more generally. Willingness to take on the task of DRE was identified as a prerequisite for efficient nurse-led practice and could lead to less reliance on urologist input.

The findings from this preliminary evaluation offer an opportunity for the ProtecT AM model of care to be developed further. Current NICE guidelines are based on expert consensus in relation to monitoring men with localised PCa,^1^ and much further research is required to determine safe and accurate triggers for disease review or the inclusion of repeat MRI/bone scans or biopsies.^6^ Further research is also needed to identify changes to the protocol when the option of radical treatment is no longer required or appropriate.

## Conclusion

This study showed that the ProtecT trial nurse-led AM for men with localised PCa was acceptable to patients, urologists and nurses because of the quality and continuity of care it delivered and perceptions of cost-effectiveness. Nurse-led care requires a protocol, effective communication between nurses and urologists and for the skill mix/knowledge of nurses to develop in line with demands of role. Nurse-led care has the potential to enable more cost-effective use of resources for routine follow-up of men with PCa following AS/AM.
